# Association between antibody-dependent cellular cytotoxicity activity of antitumor antibodies and infusion reactions: a pharmacovigilance analysis using the Japanese adverse drug event report database

**DOI:** 10.1186/s40780-025-00481-y

**Published:** 2025-08-07

**Authors:** Yusuke Tabuchi, Masayuki Tsujimoto, Yuna Kise, Tomomi Sakamoto, Miyu Matsuda, Tadashi Kosaka, Kohshi Nishiguchi

**Affiliations:** 1 Laboratory of Clinical Pharmacy, Kyoto Pharmaceutical University, 5 Misasagi-Nakauchi-cho, Yamashina-ku, Kyoto, Japan; 2 Department of Pharmacy, University Hospital, Kyoto Prefectural University of Medicine, 465 Kajii-cho, Kawaramachi-Hirokoji, Kamigyo-ku, Kyoto, Japan

**Keywords:** Infusion reactions, Cytokine release, Antibody-dependent cellular cytotoxicity, Antitumor antibodies

## Abstract

**Background:**

Infusion reactions (IRs) are common adverse events associated with biologics, often triggered by cytokine release from immune and tumor cells. Antibody-dependent cellular cytotoxicity (ADCC) is a key immune mechanism in which therapeutic antibodies recruit immune effector cells, such as natural killer cells, to induce target cell lysis. This study aimed to clarify the association between activated ADCC and IRs in antitumor antibodies using the Japanese Adverse Drug Event Report (JADER) database.

**Methods:**

Data from the JADER database (April 2004 to February 2022) were analyzed to identify IRs related to 25 antitumor antibodies. The reporting odds ratio (ROR) was calculated to assess the association between IRs and antitumor antibodies, and statistical analyses were conducted using Fisher’s exact test.

**Results:**

Avelumab was the only anti-PD-1/PD-L1 antibody with a significant association with IRs (ROR: 5.44, 95% confidence interval [CI]: 3.59–8.22). Antibodies with ADCC activity detected significantly more IR signals (9/14, 64.3%) than those without ADCC activity (1/11, 9.1%; *p* = 0.0119).

**Conclusions:**

This study suggests an association between ADCC and the occurrence of IRs in antitumor antibodies based on JADER database analysis. These findings provide valuable insights for the prevention and management of IRs.

## Background

Infusion reactions (IRs) are adverse events (AEs) that frequently occur when biologics are administered, often presenting with symptoms such as fever and chills. IRs associated with biologic administration commonly manifest during initial administration. These reactions are considered acute-phase reactions triggered by cytokines released from tumor cells and effector cells that accumulate around the tumor [1]. These cytokines have been associated with various symptoms, including fever, chills, hypotension, and dyspnea. Severe IR has been reported in some patients receiving biologics [2]. Monoclonal antibodies are classified into four types: mouse, chimeric, humanized, and fully human antibodies. The proportion of heterologous protein derivatives decreases in the listed order, correlating with a higher incidence of IRs [3]. Notably, the incidence of IRs for the humanized antibodies bevacizumab and panitumumab is low, at 1.6% [4] and 1.5% [5], respectively. In contrast, the humanized antibodies obinutuzumab and alemtuzumab showed a high incidence of IRs at 59.3% [6] and 97.4% [7], respectively. These findings indicate that the incidence of IRs varies widely, even among humanized antibodies, suggesting that the risk of IRs cannot be explained solely by the degree of humanization during antibody preparation.

The incidence of IRs varies widely, depending on the drug. Identifying which drugs are more likely to cause IRs remains challenging, as multiple factors, including patient background, influence their occurrence in clinical practice. We have demonstrated that high lymphocyte blood levels are a risk factor for IRs in patients receiving trastuzumab plus pertuzumab therapy for breast cancer [8]. Pertuzumab and trastuzumab activate antibody-dependent cellular cytotoxicity (ADCC) through effector cells such as macrophages and natural killer (NK) cells [9]. ADCC is an immune response in which antibodies bind to cancer cells, attracting immune cells, such as NK cells, to attack and injure the cancer cells. Activated NK cells, a type of lymphocyte, can produce cytokines; therefore, we considered that higher numbers of NK cells release more cytokines, potentially increasing the incidence and severity of IRs.

Among anti-PD-1/PD-L1 antibodies, only avelumab is recommended for premedication with antihistamines and acetaminophen [10]. The reported incidence of IRs associated with avelumab, which has ADCC activity, ranges from12.2 to 17.0% [10, 11], higher than that of nivolumab (1.6%) [12] and durvalumab (1.7%) [13], both of which lack ADCC activity. We hypothesized that antitumor antibodies with ADCC activity are more likely to cause IRs. Clarifying this hypothesis will facilitate the prevention and management of IRs. A systematic review and meta-analysis of high-quality phase III trial data would also be a valid approach. However, such trials may not report the incidence of IRs as seen in the phase III trial of trastuzumab. Moreover, strict inclusion criteria often exclude key patient populations, such as elderly or those with poor performance status. In this study, the JADER database was selected for this study because it captures adverse events from a broader and more diverse patient population encountered in real-world clinical practice.

The purpose of this study was to clarify the association between activated ADCC and IRs in antitumor antibodies using the JADER database.

## Methods

### Study design

Data recorded from April 2004 to February 2022 from the JADER database were downloaded from the Pharmaceuticals and Medical Devices Agency (PMDA) website (http://www.pmda.go.jp/). The JADER dataset consists of four tables containing the following data: (1) patient information, including sex, age, and weight; (2) patient drug information; (3) AEs and outcomes; and (4) medical history and primary disease. These four tables were integrated using Power BI (Microsoft^®^). Based on the data on administered drugs, the drugs associated with AEs were categorized into three groups: “suspected drug,” “concomitant drug,” and “interaction.” In this study, we examined only drugs classified as “suspected drug” in order to focus on those with a strongly suspected causal relationship. Intravenous formulations were included, whereas oral formulations were excluded.

### Extraction of target drugs

This study included 25 antitumor antibodies approved for therapy in Japan. The presence or absence of ADCC activity in each drug was determined based on package inserts and published articles. Avelumab, cetuximab, daratumumab, elotuzumab, isatuximab, mogamulizumab, obinutuzumab, pertuzumab, polatuzumab vedotin, rituximab, and trastuzumab were determined based on package inserts [14–24]. Alemtuzumab, atezolizumab, bevacizumab, blinatumomab, brentuximab vedotin, durvalumab, gemtuzumab ozogamicin, inotuzumab ozogamicin, ipilimumab, nivolumab, panitumumab, pembrolizumab, ramcirumab, and trastuzumab emtansine were determined based on articles [25–35]. 

### Definition of AEs and underlying disease

We used the International Council for Harmonisation of Technical Requirements for Pharmaceuticals for Human Use (ICH) Medical Dictionary for Regulatory Activities (MedDRA) version 24.1 to extract AEs listed in the JADER database. AEs were defined as those recorded in each patient’s AEs and outcomes tables. IRs were defined as “infusion-related reaction (PT:10051792)” and “cytokine release syndrome (PT:10052015)” according to previous article [36]. 

### Statistical analyses

The ROR is calculated as the odds of reporting a specific AE caused by a particular drug divided by the odds of reporting the same AE for all other drugs in the database. This ratio is used to identify imbalances in drug safety data and evaluate the connection between certain drugs and specific AEs. We calculated the ROR using a two-by-two contingency table to detect potential associations between antibodies and IRs. A signal was considered positive when the lower limit of the 95% confidence interval (CI) of the ROR was greater than 1. Two or more cases were required to define a signal. The association between IRs and ADCC activity or antibody type was compared using Fisher’s exact test. To account for the multiple statistical tests performed, we applied the Benjamini–Hochberg procedure to adjust *p*-values and reduce the likelihood of false-positive findings. The significance level was set at *p* < 0.05 for all comparisons. All statistical analyses were conducted using EZR [37], a modified version of the R Commander interface in R software designed to add statistical functions frequently used in biostatistics.

### Volcano plot

A volcano plot displaying the relationship between ROR and *p*-values for all drugs was generated. The natural logarithm of the ROR (lnROR) and the negative logarithm of the *p*-value (-log *p*-value) were used to construct the scatterplot. Drugs located in the upper-right area of the scatterplot indicated a close association between the occurrence of IRs and ADCC activity.

## Results

### Signals of IRs with ROR in anti-PD-1/PD-L1 antibodies

From April 2004 to February 2022, 97,567 cases of intravenous biologic infusions were analyzed in this study. Among these, 1762 cases exhibited IRs. Table 1 presents signals related to IRs detected in patients receiving avelumab among anti-PD-1/PD-L1 antibodies (ROR: 5.44, 95% CI: 3.59–8.22). No signal was detected for other anti-PD-1/PD-L1 antibodies.

**Table 1:** Signals of infusion reactions and presence of Antibody-Dependent cellular cytotoxicity in anti-PD-1/PD-L1 antibodies

	Classification	Antibody	IRs	AE	No IR	ROR	95% CI
Avelumab	Anti-PD-L1	Fully human	25	278	253	5.44*	3.59–8.22
Durvalumab	Anti-PD-L1	Fully human	9	2851	2842	0.17	0.09–0.32
Atezolizumab	Anti-PD-L1	Humanized	33	3894	3861	0.45	0.32–0.64
Nivolumab	Anti-PD-1	Fully human	142	18,246	18,104	0.38	0.32–0.45
Pembrolizumab	Anti-PD-1	Humanized	46	11,716	11,670	0.19	0.14–0.26

*Indicates statistically significant signals

IR: Infusion reaction; CI: Confidence Interval; AE: Adverse event; ROR: Reporting odds ratio

### IR signal detection with and without ADCC activity

Table 2 presents the ROR values for reports classified based on the ADCC activity of the target drugs. A signal was detected in the group with ADCC activity (ROR: 4.39, 95% CI: 3.98–4.85), whereas no signal was detected in the group without ADCC activity (ROR: 0.16, 95% CI: 0.14–0.18).

**Table 2:** Detection of infusion reaction signals with and without antibody-dependent cellular cytotoxicity

ADCC activity	IRs	AE	No IR	ROR	95% CI
ADCC positive	1,142	29,459	28,317	4.39*	3.98–4.85
ADCC negative	450	65,663	65,213	0.16	0.14–0.18

*Indicates statistically significant signals

ADCC: Antibody-dependent cellular cytotoxicity; IR: Infusion reaction; CI: Confidence Interval; AE: Adverse event; ROR: Reporting odds ratio

### Influence of ADCC activity and antibody type on IR signal detection

**Fig. 1:**
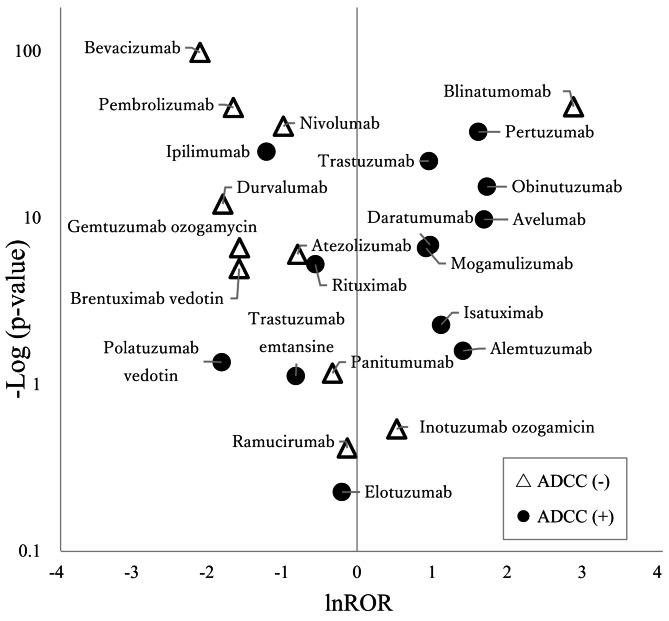
Strength of association with infusion reactions (volcano plot). Cetuximab had a *p*-value of zero and was therefore excluded from this figure

Table 3 presents the ROR values and ADCC activity of the target drugs, indicating a tendency for signal detection in drugs with ADCC activity. Table 4 shows that drugs with ADCC activity (9/14, 64.3%) detected significantly more IR signals than those without ADCC activity (1/11, 9.1%; *p* = 0.0119). Table 5 shows that no specific antibody type exhibited significant IR signals (*p* = 0.677). Figure 1 illustrates the relationship between the *p*-value and ROR for each antibody. Drugs with ADCC activity are predominantly positioned on the right side of the plot, whereas those without ADCC activity are mainly on the left side.

**Table 3:** Reporting odds ratio of infusion reactions with and without antibody-dependent cellular cytotoxicity in 25 drugs

	Antibody	IRs	AE	ROR	95% CI	ADCC activity
Blinatumomab	Mouse	59	244	17.91*	13.31–24.10	Negative
Brentuximab vedotin	Chimeric	5	1295	0.21	0.09–0.50	Negative
Cetuximab	Chimeric	591	4799	10.99*	9.90–12.19	Positive
Isatuximab	Chimeric	9	169	3.07*	1.57–6.02	Positive
Rituximab	Chimeric	64	5958	0.57	0.45–0.74	Positive
Alemtuzumab	Humanized	4	57	4.11*	1.49–11.37	Positive
Atezolizumab	Humanized	33	3894	0.45	0.32–0.64	Negative
Bevacizumab	Humanized	53	19,237	0.12	0.09–0.16	Negative
Elotuzumab	Humanized	12	807	0.82	0.46–1.45	Positive
Gemtuzumab ozogamycin	Humanized	7	1794	0.21	0.10–0.44	Negative
Inotuzumab ozogamicin	Humanized	3	99	1.70	0.54–5.37	Negative
Mogamulizumab	Humanized	44	1011	2.51*	1.85–3.41	Positive
Obinutuzumab	Humanized	39	421	5.65*	4.05–7.89	Positive
Pembrolizumab	Humanized	46	11,716	0.19	0.14–0.26	Negative
Pertuzumab	Humanized	96	1179	5.04*	4.07–6.24	Positive
Polatuzumab vedotin	Humanized	1	329	0.17	0.02–1.18	Positive
Trastuzumab	Humanized	155	3568	2.61*	2.21–3.09	Positive
Trastuzumab emtansine	Humanized	5	616	0.44	0.18–1.07	Positive
Avelumab	Fully human	25	278	5.44*	3.59–8.22	Positive
Daratumumab	Fully human	42	916	2.65*	1.94–3.63	Positive
Durvalumab	Fully human	9	2851	0.17	0.09–0.32	Negative
Ipilimumab	Fully human	55	9351	0.30	0.23–0.39	Positive
Nivolumab	Fully human	142	18,246	0.38	0.32–0.45	Negative
Panitumumab	Fully human	36	2724	0.72	0.52–1.01	Negative
Ramucirumab	Fully human	57	3563	0.88	0.67–1.15	Negative

*Indicates statistically significant signals

ADCC: Antibody-dependent cellular cytotoxicity; IRs: Infusion reactions; CI: Confidence Interval; AE: Adverse event; ROR: Reporting odds ratio

**Table 4:** Comparison of infusion reaction signal detection with and without antibody-dependent cellular cytotoxicity

ADCC activity	IR signal detected	IR signal not detected
ADCC positive	9	5
ADCC negative	1	10

Fisher’s exact test: *p* = 0.0119

ADCC: Antibody-dependent cellular cytotoxicity; IR: Infusion reaction

**Table 5:** Comparison of infusion reaction signal detection by antibody type

	Mouse	Chimeric	Humanized	Fully human
IR signal detected	1	2	5	2
IR signal not detected	0	2	8	5

Fisher’s exact test: *p* = 0.667

IR: Infusion reaction

## Discussion

This study found that among anti-PD-1/PD-L1 antibodies, only avelumab exhibited an IR signal, indicating that ADCC activity may be associated with IR occurrence. Furthermore, antitumor antibodies with ADCC activity detected significantly more IR signals than those without ADCC activity. These findings suggest a potential association between ADCC activity and IRs.

This study showed that ADCC activity may be associated with IRs induced by antibodies (Table 3). Previously, we reported that high lymphocyte count is a risk factor for IRs in trastuzumab plus pertuzumab therapy [8]. We believe that elevated lymphocyte levels—including macrophages and NK cells involved in ADCC activity—may contribute to increased cytokine release. We considered the ADCC activity to be related to IRs.

Among anti-PD-L1 antibodies, avelumab has been reported to have a higher incidence of IRs [10, 11]. Notably, avelumab is the only anti-PD-L1 antibody for which premedication with antihistamines and acetaminophen is recommended at the time of therapy [10]. Other anti-PD-L1 antibodies, such as atezolizumab and durvalumab, have been reported to undergo glycosylation of the Fc region to eliminate the effects of ADCC activity [38]. In contrast, avelumab lacks glycosylation in the Fc region and induces ADCC by binding to the Fc region on NK cells and macrophages [39]. This suggests that modification of the Fc region is associated with ADCC activity and may be linked to the incidence of IRs.

Ipilimumab is an immune checkpoint inhibitor that targets CTLA-4 and is used in combination with nivolumab. Although ipilimumab has ADCC activity, no signal was detected in this study (Table 3). Ipilimumab reduces Treg cells via ADCC with macrophages, which in turn attracts CD8 T cells and enhances the antitumor effect [40]. However, other studies have reported that ipilimumab does not significantly reduce Treg cells of tumors in patients and exhibits low ADCC activity [41]. These findings suggest that Treg cell reduction and ADCC activity in ipilimumab may vary depending on the context and tumor microenvironment, which may explain the lack of signal detection in this study.

Blinatumomab was the only antibody without ADCC activity that appeared in the upper-right area of the volcanic plot (Fig. 1). Blinatumomab is a novel bispecific antibody, also known as Bispecific T cell Engager (BiTE), which links CD19-positive B cells to CD3-positive T cells. Thus, blinatumomab leads to B cell depletion and dose-dependent cytokine elevation [42]. Therefore, although blinatumomab does not have ADCC activity, its signal may have been detected due to its unique mechanism of cytokine release pathway.

A higher incidence of IRs has been reported for antibodies with a greater proportion of heterologous proteins, [3]; however, this trend was not observed in this study (Table 5). This discrepancy may be attributed to advances in Fc region modification technology, as seen in antibodies such as atezolizumab and durvalumab. Additionally, mogamulizumab is a humanized anti-CCR4 monoclonal antibody with a defucosylated Fc region that significantly enhances ADCC by increasing its binding affinity to effector cells ( [43]). In obinutuzumab, glycosylation of the Fc region increases the affinity of the antibody for monocytes, macrophages, and neutrophils, enhancing antibody-dependent cell-mediated phagocytic activity ( [44]). This process induces tumor cell phagocytosis, resulting in antitumor effects. We propose that advancements in glycosylation technology in the Fc region have reduced the influence of antibody type on the incidence of IRs.

The concomitant use of steroids, antihistamines, and antipyretic analgesics is recommended to prevent the occurrence of IRs [7, 10]. For the cases listed in Table 2, where IRs occurred in relation to the presence or absence of ADCC activity, we investigated the use of concomitant medications that may influence IRs. ADCC-positive cases tended to have higher rates of concomitant medication use compared to ADCC-negative cases: steroids (61.2% vs. 52.0%), antihistamines (47.1% vs. 20.0%), and antipyretic analgesics (19.4% vs. 14.2%). Although the use of these medications was more frequent in ADCC-positive cases, IR signals were still detected. In contrast, in ADCC-negative cases, the concomitant use of medications likely did not prevent the occurrences of IRs.

Few studies in Japan have examined these associations using spontaneous reporting databases. Therefore, the findings of this study should serve as a valuable reference for future considerations.

This study has some limitations. Although JADER is capable of detecting adverse effect signals, statistical limitations exist because only cases involving AEs are reported, with no control group for comparison [45]. Additionally, Adverse events reporting typically peaks within the first two years after a drug’s approval and then declines sharply over time, a phenomenon known as the Weber effect [46]. Although all antibodies analyzed in this study have been on the market for more than three years, the Weber effect may still have influenced the reporting trends. The primary reporters in JADER are physicians and pharmaceutical companies leading to reporting biases influenced by market trends such as product popularity and timing of sales. This study evaluated each drug individually and did not assess the impact of concomitant antibody use. Ipilimumab has been reported to increase the incidence of CRS when combined with nivolumab [47], and the combination of trastuzumab with pertuzumab has been associated with increased severity of IRs [48]. Further research is needed to clarify how antibody combinations influence the occurrence and severity of IRs. This study used data only from Japan, further research is needed when applying the results to other countries or populations with different ethnic backgrounds.

## Conclusions

This study suggests an association between ADCC activity and IR occurrence in antitumor antibodies based on the JADER database analysis. These findings provide valuable insights for the prevention and management of IRs and may guide the safer clinical application of antitumor antibodies.

## Data Availability

No datasets were generated or analysed during the current study.

## Abbreviations

-log *p*-value: Negative logarithm of the *p*-value
ADCC: Antibody-dependent cellular cytotoxicity (ADCC)
AE: Adverse event
BiTE: Bispecific T cell Engager
CI: Confidence Interval
ICH: International Council for Harmonisation of Technical Requirements for Pharmaceuticals for Human Use
IR: Infusion reaction
JADER: Japanese Adverse Drug Event Report
lnROR: Natural logarithm of the reporting odds ratio
MedDRA: Medical Dictionary for Regulatory Activities
NK: Natural killer
PMDA: Pharmaceuticals and Medical Devices Agency
ROR: Reporting odds ratio
